# Thermally-drawn fibers with spatially-selective porous domains

**DOI:** 10.1038/s41467-017-00375-0

**Published:** 2017-08-28

**Authors:** Benjamin Grena, Jean-Baptiste Alayrac, Etgar Levy, Alexander M. Stolyarov, John D. Joannopoulos, Yoel Fink

**Affiliations:** 1 Research Laboratory of Electronics, Massachusetts Institute of Technology, Cambridge, MA 02139 USA; 2 Department of Materials Science and Engineering, Massachusetts Institute of Technology, Cambridge, MA 02139 USA; 3 Institute for Soldier Nanotechnologies, Massachusetts Institute of Technology, Cambridge, MA 02139 USA; 4 MIT Lincoln Laboratory, Lexington, MA 02421 USA; 5 Department of Physics, Massachusetts Institute of Technology, Cambridge, MA 02139 USA

## Abstract

The control of mass transport using porous fibers is ubiquitous, with applications ranging from filtration to catalysis. Yet, to date, porous fibers have been made of single materials in simple geometries, with limited function. Here we report the fabrication and characterization of thermally drawn multimaterial fibers encompassing internal porous domains alongside non-porous insulating and conductive materials, in highly controlled device geometries. Our approach utilizes phase separation of a polymer solution during the preform-to-fiber drawing process, generating porosity as the fiber is drawn. Engineering the preform structure grants control over the geometry and materials architecture of the final porous fibers. Electrical conductivity of the selectrolyte-filled porous domains is substantiated through ionic conductivity measurements using electrodes thermally drawn in the cross-section. Pore size tunability between 500 nm–10 µm is established by regulating the phase separation kinetics. We further demonstrate capillary breakup of cylindrical porous structures porous microspheres within the fiber core.

## Abstract (web summary)

Porous polymer fibers show great potential for a range of applications, but their simple structures typically limit their functionality. Here, the authors combine a thermal drawing process with polymer solution phase separation to fabricate porous multimaterial fibers with complex internal architectures.

## Introduction

Porous polymeric fibers are high aspect ratio filaments displaying a substantial level of internal porosity. Such fibers find applications in gas separation^1–3^, water filtration^4–6^, hemodialysis^7–9^, and tissue engineering^10–12^. The fabrication of these fibers generally relies on the extrusion—or spinning—of a polymer solution followed by its phase separation^13^. In particular, in the case of thermally induced phase separation (TIPS), a solution is homogenized at high temperature and cooled or quenched so as to trigger its phase separation into a solvent-rich and polymer-rich phase with subsequent solidification of the polymer-rich phase^14, 15^. The solvent can later be removed by rinsing or evaporation to yield the final porous structure. With this process, porous fibers from a variety of polymers have been produced^16–20^; however, they are generally made from a single material in a cylindrical geometry and are intrinsically passive. In this study, we report on the fabrication of complex fiber architectures with multiple materials organized around a porous domain, paving the way towards new types of active multifunctional porous fiber devices, such as flow-sensing filtration fibers, sweat sensing textiles or electrically active cell scaffolds.

The preform-to-fiber thermal drawing method has proven to be a versatile process allowing the fabrication of polymer-based fibers with complex multimaterial internal structures^21–28^. Thermally drawn fibers can include a wide range of materials including metals, semiconductors, composites or ferroelectric polymers, granting them capabilities such as light sensing^22, 24^, piezoelectric actuation^25, 26^, chemical detection^28^ and neural activity stimulation and recording^29, 30^. However, the fabrication of fibers with internal porous domains through this method has remained elusive. The challenge arises from the fabrication method itself, which relies on the flow and elongation of low-viscosity fluids. Direct incorporation of porous materials in the preform results in elongated hollow domains in some cases and collapsed domains in others.

Here we present an approach combining the advantages of thermal drawing with the principle of polymer solution thermally induced phase separation (TIPS) to generate porous fibers with complex architectures that could not be achieved otherwise. For this we take advantage of the fact that the thermal drawing method intrinsically takes the preform materials from a high temperature state in the furnace, to a fiber-form low-temperature state upon exiting the furnace. Therefore, by filling the preform with a well-chosen polymer solution, we can trigger the latter’s phase separation during the draw and produce complex multimaterial fibers with porous domains enabling inter- and intra-fiber transport.

## Results

### Porosity generation by TIPS

**Fig. 1:**
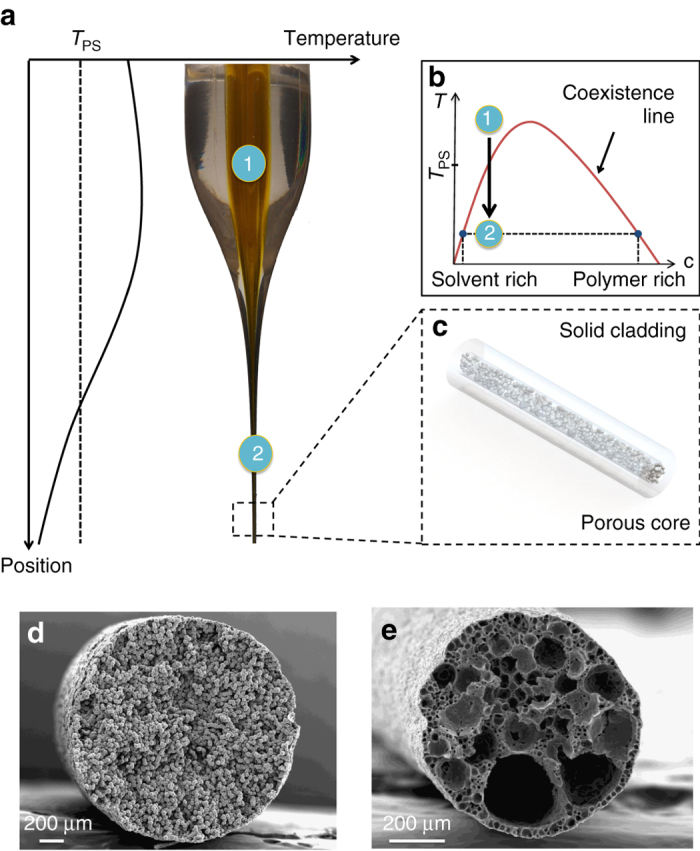
Fiber fabrication method and cross-sections. (**a**) General illustration of thermal drawing process, with the associated temperature profile. The dashed line denotes the phase separation temperature for the polymer solution in the core. (**b**) Schematic phase diagram for a generic polymer solution. State 1 is the homogeneous state in the furnace and state 2 is the phase separated state at room temperature. (**c**) Illustration of a section of drawn fiber with the dense cladding surrounding the porous core. (**d**, **e**) Cross-sectional SEM images of porous fibers made of (**d**) polyvinylidene fluoride and (**e**) polycaprolactone (PCL) obtained after cladding dissolution

The fiber fabrication method starts with the assembly of a macroscopic object called a preform^31^, which serves as a template for the porous fiber we wish to produce. The preform acts here as a reservoir with a well-defined architecture that is filled with a polymer solution before the draw. During the drawing process, the preform is heated in a furnace above the glass transition of the preform materials and above the phase separation temperature of the polymer solution, such that the solution remains homogeneous in the furnace. The preform is drawn into a fiber by constant pulling at a controlled speed, and the fiber cools down to room temperature upon exiting the furnace (Fig. 1a). Provided the polymer solution demixes between the drawing temperature and ambient temperature, the solution phase separates in the fiber leading to a porous polymeric core whose pores are filled with a solvent-rich phase (Fig. 1c), much like in a conventional solution extrusion technique. Phase separation in TIPS processing can either occur through liquid–liquid (L–L) demixing^15^, where the two phases initially formed are liquid and the polymer-rich phase solidifies at a lower temperature; or solid–liquid (S–L) demixing in which case the polymer crystallizes out of the solvent directly^14^. In this work, we exploit both mechanisms. As an illustrative example, Fig. 1b shows a typical phase diagram for a solution which phase separates via a L–L demixing mechanism^15, 32^. After the draw, the dense cladding illustrated in Fig. 1c may be dissolved with an appropriate solvent and the liquid phase subsequently evaporated, resulting in porous fibers.

We demonstrate the process using a cyclic olefin copolymer (COC, TOPAS 8007 from TOPAS Advanced Polymers) preform cladding as a cylindrical reservoir. COC is an amorphous thermoplastic displaying excellent chemical resistance to many polar organic solvents, and can be thermally drawn between 150 to 280 °C. Before the draw, a cylindrical hollow reservoir is filled with a 1 : 4 wt. solution of polyvinylidene fluoride (PVdF, Kynar 761, Arkema) in propylene carbonate. This solution has a S–L demixing temperature around 35 °C^33^, so that it is homogeneous during the draw process but phase separates in the fiber core as it cools down to ambient temperature. Porous PVdF fibers are obtained by selectively dissolving the COC cladding in toluene. The scanning electron microscopy (SEM) micrograph of the fiber cross-section (Fig. 1d) shows a morphology consisting of interconnected PVdF spherulites separated by voids. Such morphology is common for S–L phase separated PVdF membranes^14, 32^, and arises from nucleation and growth of highly semicrystalline PVdF spherulites^33^.

This method can be leveraged to generate porous fibers of different materials. As another example, a solution of poly-ε-caprolactone (PCL, Mn≈80 k, Sigma-Aldrich) in a 1 : 1 wt. mixture of propylene carbonate and triethylene glycol was added to a COC-clad preform. These two miscible solvents act as a latent solvent for the polymer and a phase separation of the solution also occurs in the fiber as it cools down to room temperature. A SEM micrograph of the porous core is shown in Fig 1e. It displays a very different morphology than that of the porous PVdF fibers, with a broad distribution of cellular pores. Supplementary Fig. 1 shows the result of a dye diffusion experiment proving the existence of a continuous diffusion path along the pores—the details of the experiment are outlined in Supplementary Note 1. This morphology is indicative of a L–L phase separation occurring through nucleation of a solvent-rich phase followed by subsequent solidification of the polymer-rich phase^15, 32^ and has also been reported previously in literature for PCL membranes^34^.

### Control over geometry and materials architecture

**Fig. 2:**
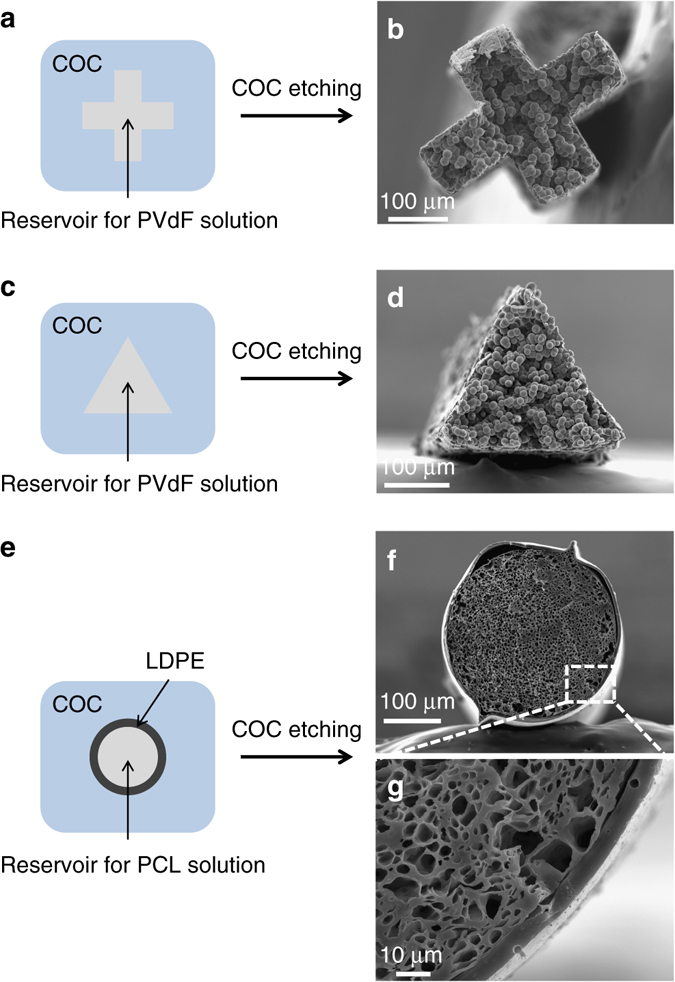
Control of external geometry and architecture. (**a**) Schematic illustration of a preform with a cross-shaped reservoir for PVdF solution and (**b**) SEM micrograph of the cross-shaped porous PVdF fiber after drawing and cladding dissolution. (**c**) Schematic illustration of a preform with a triangle-shaped reservoir for PVdF solution and (**d**) SEM micrograph of the triangle-shaped porous PVdF fiber after drawing and cladding dissolution. (**e**) Schematic illustration of a cyclic olefin copolymer preform with a cylindrical reservoir for polycaprolactone (PCL) solution lined with a thin LDPE wall and (**f**, **g**) SEM micrographs of the final porous PCL core/dense LDPE shell fiber after drawing and cladding removal

This preform-to-fiber TIPS processing methodology is characterized by a number of unique attributes. The first advantage is that we can easily control the fiber cross-sectional geometry, unlike in fiber extrusion-based processes which requires complex spinneret engineering as well as optimized cooling and solidification conditions^35^. The preform acts here as a template for the porous fiber, and by machining the internal geometry of the preform into arbitrary shapes, we can produce porous fibers with complex external geometries such as triangular, or cross-shaped (Fig. 2a–d). The ability to do so is a consequence of the large viscosity contrast between the cladding and the solution during the drawing process. At the high draw temperature, the cladding material exhibits a viscosity close to 10^4^ Pa s^−1^, roughly four to five orders of magnitude higher than that of the polymer solution (see Supplementary Note 2 and Supplementary Fig. 2). This high viscosity of the cladding kinetically prevents the capillary rounding of sharp features to occur, whereas the low-viscosity solution fills the whole accessible volume and phase separates into a porous fiber of the same shape (cf. Supplementary Methods and Supplementary Figs 5 and 6 for more details on the drawing process). The external geometry of the porous fiber is therefore only limited by our ability to machine a preform of the matching shape, and does not require complex spinneret engineering and optimization of spinning conditions. Even with advanced spinneret design and spinning processes, obtaining geometries that significantly deviate from equilibrium still presents significant challenges^35^. Controlling the external geometry could enable control over the mechanical properties of the fibers, as was shown for natural jute fibers for instance^36^.

The second and most important key benefit of the preform-to-fiber TIPS is the ability to combine multiple materials adjacent to a porous domain. In solution extrusion-based methods, this requires the design of advanced concentric co-extrusion nozzles. Here, multimaterial structural control is achieved by initially designing and assembling a preform template with the desired materials. We demonstrate this by producing a porous PCL core/dense low-density polyethylene (LDPE) shell fiber, using our preform-to-fiber TIPS approach (Fig. 2e–g). First, a preform is constructed consisting of a hollow core lined by a shell of LDPE of 200 µm, embedded in a large centimer-size COC cladding (Fig. 2e). Second, the PCL solution is introduced into the hollow core and the multimaterial preform is drawn into a fiber. Finally, the COC cladding is selectively dissolved using cyclohexane and the solvent is dried out of the core to leave only a thin shell of LDPE surrounding a porous PCL core (Fig. 2f–g). Here, the in-fiber LDPE shell wall thickness is 10 µm, but in principle it can be made thinner by faster drawing speeds. This type of concentric core-shell cylindrical structure could be useful for gas separation fibers with dense selective barriers^2, 3^.

### Pore size tunability through kinetics of phase separation

**Fig. 3:**
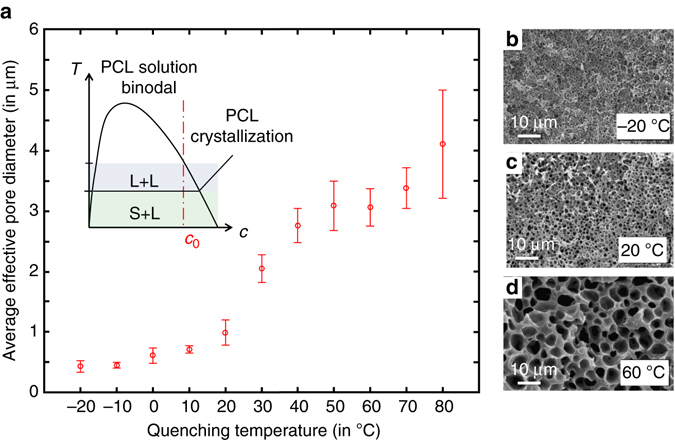
Influence of quenching temperature on microstructure. (**a**) Average pore size for different quenching temperatures in ethylene–glycol–water bath. The error bar corresponds to the SD over the mean pore size in the six images analysed per quenching conditions. The inset displays an illustration of the possible phase diagram with a dashed line at the working concentration, highlighting both L–L and S–L demixing. (**b**–**d**) Associated cross-sectional SEM images for fibers quenched at −20, 20 and 60 °C

In addition to external geometry and materials distribution, the preform-to-fiber TIPS method also allows some degree of control over the microstructure of the porous domain through control of the quenching temperature, specifically for L–L demixing solutions. Figure 3 demonstrates the effects of fiber quenching temperature on the average pore size of a PCL porous core fiber, estimated from an image analysis of the cross-sectional micrographs. For this study, fiber samples were reheated post-draw to *T*_reheat_ = 150 °C in order to rehomogenize the solution, and quenched for a fixed time Δ*t* = 5 min in a water/ethylene glycol bath at set temperatures *T*_bath_. Three fiber samples were post-processed per quenching temperature, and a total of 6 SEM micrographs were analysed per cooling condition. The results indicate that fibers quenched in colder baths exhibit smaller average pore sizes, with an apparent transition in the behaviour of pore size vs. quenching temperature around 40 °C.

These results can be rationalized considering the specifics of the PCL solution’s phase diagram, schematically represented in the inset of Fig. 3a. At our working concentration, the solution exhibits a L–L demixing at a relatively elevated temperature (≈110 °C, observed visually by appearance of turbidity in the solution), followed by a PCL solidification at lower temperatures, around 40 °C (also observed qualitatively). Therefore, when quenched above 40 °C, we expect the L–L phase separated structure to exhibit coarsening. During coarsening, the system seeks to minimize the interfacial energy between the polymer-rich phase and the solvent-rich phase by minimizing the surface area and forming larger voids^32, 37^. This can happen either through diffusion of molecules from small to large droplets (Ostwald ripening), coalescence of droplets or hydrodynamic flow^38, 39^. All these processes result in scaling laws of the form:

1 $${\left\langle {R} \right\rangle}^\alpha = {\left\langle {{R_0}} \right\rangle ^\alpha } + {K_\alpha }\left( T \right){\rm{\Delta }}t$$

where *α* is 3 for Ostwald ripening or coalescence-based coarsening and 1 for coarsening through hydrodynamic flow, *K*_α_ is a prefactor depending on temperature and on the exact mechanism involved, and *R*_0_ is the average droplet nucleus size^38, 39^. Although the exact functional for *K*_α_ is unknown for our system, all of the associated processes are thermally activated and thus their rates grow with increasing temperature—explaining the increase in pore size with temperature. For samples quenched in a bath below 40 °C, the PCL will rapidly crystallize, thus setting the structure and preventing further coarsening. The effective time of coarsening Δ*t*_eff_, or time during which the structure is in a L–L state, is reduced as the sample is convectively cooled from *T*_reheat_ to *T*_bath_. At deep enough quenching, coarsening is almost completely suppressed and the average pore size approaches the droplet nucleus size, setting a lower limit on pore size for L–L demixing solutions. For this reason, the pore size is nearly temperature independent for deep quenching as seen in Fig. 3a. The Supplementary Discussion gives more details on the coarsening behaviour.

In practice, this cooling step could be added in series with the drawing process itself to permit direct control of the pore size along arbitrary lengths of fiber. The coarsening mechanism varies between solution systems, specific concentrations, temperatures and time—but *α* and *K*_α_ can be determined experimentally by performing measurements of the average pore size with time at fixed temperatures^39, 40^. This knowledge could then be used to determine specific cooling conditions required to obtain a desired pore size.

### Multimaterial fiber for transverse ionic conductivity measurements

So far we have demonstrated the ability with our method to control the microstructure as well as incorporate multiple materials adjacent to a porous domain within a fiber. These features open up the possibility of introducing functionalities in porous fibers that go beyond conventional passive mass transport. To demonstrate this idea, we developed a fiber that can be used to measure the ionic conductivity of an ionically conductive liquid filling the porous domain in the core of a fiber.

In our process, when the fiber exits the furnace and cools down, the core consists of a porous polymer filled with a solvent-rich liquid phase. By initially introducing a soluble and thermally stable ionic liquid in the core solution, the pores can be filled with an ionically conductive electrolyte in a single step. We can then use ionic conductivity measurements to probe the transport properties of the fibers. We focus here on measurements performed in the transverse direction of the fiber, whereas experiments on axial measurements are presented in Supplementary Note 3 and shown in Supplementary Fig. 3.

**Fig. 4:**
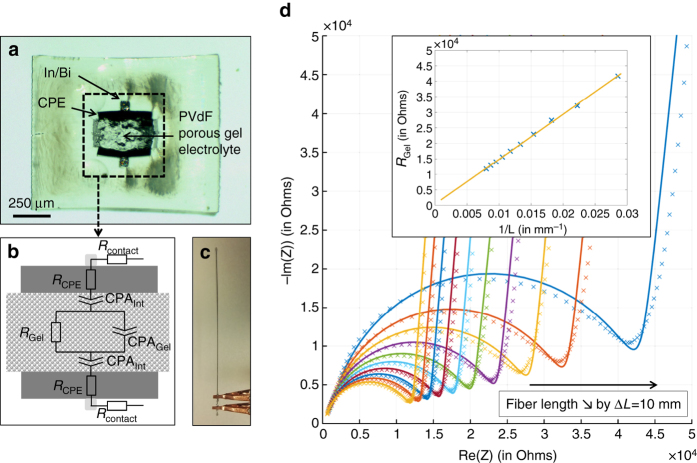
Transverse ionic transport measurement through impedance spectroscopy of ionic liquid-filled porous core fibers. (**a**) Optical micrograph of fiber sample displaying a porous core filled with a 10^−3^ m PYR13TFSI in propylene carbonate solution, adjacent CPEs and contiguous Bi-In metal buses. (**b**) Simple equivalent circuit expected from fiber samples. CPA refer to Constant Phase Angle elements. (**c**) Photograph of a connected fiber sample. (**d**) Impedance spectra for a fiber between 12 and 1 cm, sequentially cut by 10 mm decrements. Solid lines are fittings results with equivalent circuit. The inset shows the dependence of *R*_Gel_ as a function of the inverse-length of the fiber and shows a linear relationship, as expected from geometric considerations

We start by employing the preform-to-fiber TIPS method to fabricate a fiber with the cross-section shown in Fig. 4a. The core is a porous domain prepared from a solution of 1 : 4 wt. PVdF and propylene carbonate, with the addition of a 10^−3^ m (mol kg^−1^ of solvent) PYR13TFSI (Solvionic) ionic liquid in propylene carbonate providing ionic conductivity. We selected PYR13TFSI as an ionic liquid owing to its solubility in propylene carbonate and high thermal stability preventing degradation during the draw. A low ionic liquid concentration was intentionally selected to decrease the conductivity of the electrolyte and later be able to neglect resistive contributions from other elements in the system. Adjacent to the porous core are two carbon-loaded polyethylene electrodes (CPE), contiguous with two eutectic Bi-In (Indium Corporation) metal buses. The combination of a porous domain, composite electrodes, and metal buses within a single fiber is out-of-reach to standard extrusion-based processing methods for porous fibers. Here we are able to produce the fiber by initially combining the different materials in a prescribed architecture at the preform level, subsequently inserting a solution in the core, and drawing the preform into a fiber.

In-fiber ionic conductivity was measured by AC impedance spectroscopy. This method consists of measuring the impedance of a sample over a wide frequency range and mapping the results to a physically motivated equivalent circuit^41^. In our case, the simplest equivalent circuit is presented in Fig. 4b, following typical analysis of electrolyte systems. For a given fiber length, the CPE electrodes are counted for their resistive contribution through *R*_CPE_, whereas the electrode/gel-electrolyte interface is modeled as a Constant Phase Angle element $Z_{{\rm{int}}}^{{\rm{CPA}}}$. The bulk porous-electrolyte is modeled with a parallel circuit of a resistive component *R*_Gel_ associated with ion-transport in the liquid region and a capacitive component $Z_{{\rm{Gel}}}^{{\rm{CPA}}}$ in the form of a CPA element, which takes into account polarization effects in the dielectric electrolyte region. CPA elements, also known as Constant Phase Angle elements, are used in AC impedance models and data fitting to represent so-called imperfect capacitor behaviors^41^.

To perform the measurement, the fiber samples are cut to a specific length and the tips are sealed with wax to prevent solvent evaporation. Electrical connection to the fibers is established by mechanically exposing the metal buses on both sides and connecting them to the instrument (Solartron 1287 A) using copper clips (Fig. 4b). The impedance is measured over the frequency range 1 MHz–100 Hz and gradually the fiber is cut by 10 mm increments. Nyquist plots for such samples are shown in Fig. 4d, with best fits from the equivalent circuit detailed above. From this we can extract the resistance of the porous electrolyte for various lengths—in practice, close to the diameter of the high frequency semicircle. The inset shows the plot of *R*_Gel_ as a function of fiber inverse length, which follows a linear behavior expected from:

2 $${R_{{\rm{Gel}}}} = \frac{1}{{{\sigma _{{\rm{Gel}}}}}} \cdot \frac{t}{{w \cdot L}}$$

where *σ*_Gel_ is the ionic conductivity of the porous electrolyte, *t* is the thickness and *w* the width of the electrolyte region. We deduce the effective ionic conductivity of 2.87 ± 0.47 µS cm^−1^ (*n* = 5 samples) from geometric considerations, compared with 2.09 µS cm^−1^ for the pure PYR13TFSI in PC electrolyte (see Supplementary Note 4 and Supplementary Fig. 4). We attributed the discrepancy to possible inhomogeneities in the ionic liquid concentration, as well as possible solvent evaporation during the drawing process.

This experiment not only demonstrates our ability to produce complex fibers with porous domains, composite polymers and metals, but also that we are able to build functionality into the fiber. In this case we can add electrical capabilities by introducing conductive materials adjacent to the porous core and use these capabilities to get information on the electrolyte contained in the core. This capability, for example, could be applied later to smart textiles with the ability to intake and electrochemically monitor sweat.

### Porous spheres through capillary breakup

**Fig. 5:**
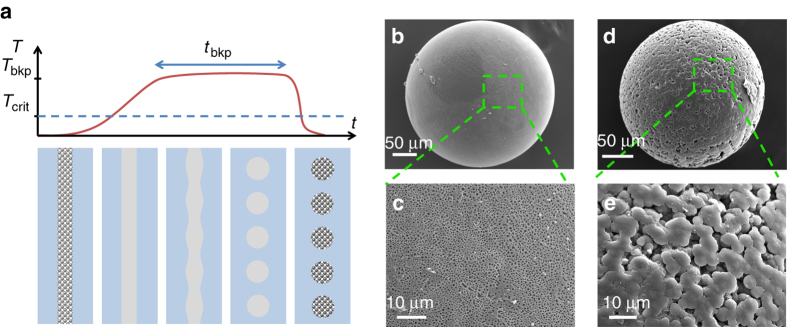
Porous microsphere production with controlled capillary breakup of porous core fibers. (**a**) General illustration of the capillary breakup process. Fibers are reheated above the phase transition temperatures of the solution and glass transition of the cladding. The core then evolves into a row of spheres under the effect of surface tension. Once breakup is completed samples are rapidly quenched. (**b**, **c**) SEM image and close-up of a polycaprolactone (PCL) porous microsphere and of (**d**, **e**) a polyvinylidene fluoride porous microsphere

Finally, the preform-to-fiber TIPS method is not only a platform for production of porous fibers, but it can also be harnessed to produce porous microspheres. To this end, we build on previously established results regarding controlled capillary instabilities in thermally drawn fibers^42–44^. After the draw, fibers (with porous core and non-porous cladding in place) are reheated above the glass transition temperature of the cladding material and critical temperature of the solution. The solution homogenized and the cylindrical core evolves into a row of particles, under the effect of capillary forces. The timescale to complete breakup is a function of the core size, viscosity of both the cladding and the core, as well as the surface tension of the core/cladding interface^45^. Depending on the sizes and processing temperature, this timescale is on the order of tens of minutes to hours (cf. Supplementary Movie 1). After the complete formation of spheres, we could quench the sample and thus obtain a fiber whose core consisted of a series of discrete porous particles, which could later be extracted by cladding dissolution. A schematic representation of this general process is shown in Fig. 5a.

By applying this method to COC-clad fibers with cores filled with either PCL or PVdF solutions, we obtained porous spherical particles of both polymers. We show SEM micrographs of both types of particles in Fig. 5b–e. Porous particles such as these could find applications in solid phase extraction or chromatography^46^. Furthermore, one could combine breakup with multimaterial porous fibers to generate structured multimaterial porous microspheres.

## Discussion

The method we introduce combines the advantages of fiber thermal drawing with the basic principle of TIPS of polymer solutions to produce unique multimaterial porous fibers. The preform design process however involves some constraints on the materials selection. In the simplest case, building a preform involves the choice of three materials: a polymer cladding and a solvent/polymer system as a solution. The set of constraints on the materials selection is the following: the solvent used should not dissolve the cladding material in the processing temperature range; the solution must not boil at the drawing temperature; and finally the solution must exhibit either a L–L or L–S separation temperature between ambient temperature and the drawing temperature.

Despite these constraints, the reported approach offers unparalleled possibilities for controlling the architecture and functionality of porous fibers. We have shown that we can dictate the external geometry of the fibers, as well as tune the porous microstructure, and most importantly associate porous domains with non-porous domains of various materials within a single fiber. The as-drawn fibers can also be post-processed to produce porous microspheres of various materials. This preform-to-fiber TIPS method provides a compelling platform for the production of new types of porous fibers and could enable a transition from passive fiber membranes to active multifunctional devices.

## Methods

### Preform fabrication, solution incorporation and fiber drawing

The preform cladding material is COC, supplied by TOPAS (grade used: 8007). Pellets are molded into slabs in aluminum molds using a vacuum oven operating at 280 °C for 10 h under constant vacuum. The slabs are then machined using either a lathe or a milling machine, depending on the desired shapes. For example, for the cross-shaped fiber, two COC slabs are milled symmetrically to introduce a half-cross pattern in each one. The two slabs are then fused in a hot press at 130 °C for 2 h, with a cross-shaped PTFE spacer filling the reservoir to prevent collapse.

PVdF solutions were prepared from PVdF (Kynar 761, provided by Arkema) and propylene carbonate (Sigma-Aldrich). PCL solutions were prepared from PCL (Mn ≈ 80,000, Sigma-Aldrich) and 1 : 1 wt. mixture of propylene carbonate and triethylene glycol (Sigma-Aldrich). All materials were used as received. The polymers and solvents were mixed to the desired ratios (1:4 wt. for PVdF, 1 : 6wt. for PCL) in a vial at 200 °C until homogeneous. Immediately before the drawing process, the solutions are poured into the preform reservoir and the top of the preform is sealed by hot-pressing a thin COC sheet.

The fiber drawing process starts at 210 °C and the drawing continues at temperatures between 150 °C and 210 °C. The preform is fed downwards into the furnace at a constant rate of 1 mm min^−1^. The fiber is pulled with a capstan wheel at a rate between 0.2 and 5 m min^−1^, depending on the processing conditions and final size objective. During the drawing process, the fiber cross-sectional dimensions and the tension in the fiber are monitored in real time.

### SEM sample preparation

Fiber samples obtained from the draw were imaged using SEM (JEOL 6010LA). For all SEM images shown, the COC cladding was etched out of the fiber post draw. The removal of the cladding was done by dissolving COC in excess cyclohexane. The porous fiber was then collected, rinsed in ethanol and dried under vacuum for 24 h before SEM observation. Samples are freeze-fractured in a bath of liquid nitrogen and gold sputtered before SEM imaging.

### Microstructure control via quenching temperature

Fibers for the microstructure control experiment were drawn in a state of low stress (<50 g mm^−2^), as measured during the drawing process by a tension-meter and laser caliper. A 10 cm-long fiber samples were then reheated on a glass slide at 150 °C for 1 min, a temperature exceeding the phase separation temperature of the inner solution. The fibers were then rapidly (<1 s) transferred into a 1 : 1wt. ethylene glycol-water bath at a controlled temperature and left in the bath for 5 min. Following quenching, the fibers were removed from the bath and left in air. The COC cladding of the fibers was then dissolved in cyclohexane and the fibers fractured in liquid nitrogen before SEM observation.

### Pore size estimation using SEM

We analysed the average pore size from SEM images. All SEM images were taken using the same brightness and contrast settings; the images were not further corrected before being analysed. All images were binarized using the same gray-level threshold of 0.8, a value arbitrarily chosen, which corresponded to qualitatively acceptable pore detection. Then all the individual pores were detected and listed, along with their pixel-size surface areas and equivalent circle diameter. The mean pore diameter for each image was then calculated and converted into microns from the scalebar of each image. A total of three samples and six total images per quenching conditions were measured and analysed in this way so as to calculate and average pore size and SD for each quenching conditions.

### AC impedance spectroscopy

The transverse AC impedance spectroscopy was performed using an impedance analyser (Solartron 1287A, Solartron Analyticals). Fibers of various lengths were cut and capped with wax to prevent solvent evaporation. The metal buses on either side of the fibers were exposed mechanically by gentle polishing. We then applied Bi-In solder to the exposed electrodes to increase the effective contact area. Fibers were connected directly using copper clips clipped in the Bi-In solder patch on either side of the fiber. Cells were run between 100 Hz and 1 MHz with an amplitude of 100 mV to obtain the Nyquist plots. The plots were fit with the Solartron ZView software, using the equivalent circuit displayed in Fig. 4b. The length and internal cross-sectional dimensions of the fibers used to deduce the ionic conductivity were measured with a caliper and an optical microscope, respectively.

### Spheres production from capillary breakup

Cylindrical core fibers with core consisting of 1 : 6 wt. PCL in triethylene glycol/propylene carbonate 1 : 1 wt. and 1 : 4 wt. PVdF in propylene carbonate were drawn in a state of low stress. Individual fiber samples of 10 cm were then placed on a glass slide on a hot plate and heated to 160 °C under a microscope. The fibers’ core spontaneously evolved into a row of spheres over a time of ≈20 min. Once breakup was completed, as monitored through the optical microscope, the fiber samples were quenched immediately in a water bath at room temperature. The COC cladding of the fiber was then dissolved in cyclohexane and the spheres are harvested and imaged using a SEM.

### Data availability

The data that support the findings of this study are available from the corresponding author upon request.

## Electronic supplementary material

Supplementary Information

Supplementary Movie 1
