# Supplementary material for: Thermally-drawn fibers with spatially-selective porous domains
Source: Nat Commun. 2017 Aug 28;8:364. doi: 10.1038/s41467-017-00375-0 (PMC5573721; doi:10.1038/s41467-017-00375-0)
Supplement: Supplementary file 1 — Supplementary Information [file 41467_2017_375_MOESM1_ESM.pdf]

Description of Supplementary Files

File Name: Supplementary Information
Description: Supplementary Figures, Supplementary Notes, Supplementary Discussion, Supplementary Methods and Supplementary References

File Name: Supplementary Movie 1
Description: Timelapse of breakup of a polycaprolactone (PCL) solution core/cyclic olefin copolymer (COC) cladding fiber. Timelapse of breakup of a core/cladding fiber made up of a core of 1:6 wt. PCL solution in propylene carbonate/triethylene glycol 1:1 wt. and a core of COC.

Supplementary Note 1: Dye diffusion experiments on PCL porous fibers

To show that PCL porous fibers displayed an open pore structure with a continuous diffusion path along the pores, we performed a transport experiment on these fibers. As-drawn fiber samples were cut, placed in vial with one tip immersed in a solution of Sudan Blue dye in PC/Triethylene glycol 1:1wt – while the other tip was coated with wax to prevent evaporation of solvent. The samples were left in place for 48hrs, and imaged under an optical microscope as shown in Supplementary Figure 1.

The ability of the dissolved dye to diffuse ≈10mm along the length of the fiber core is an indication of a continuous diffusion path in the liquid phase along the pores.

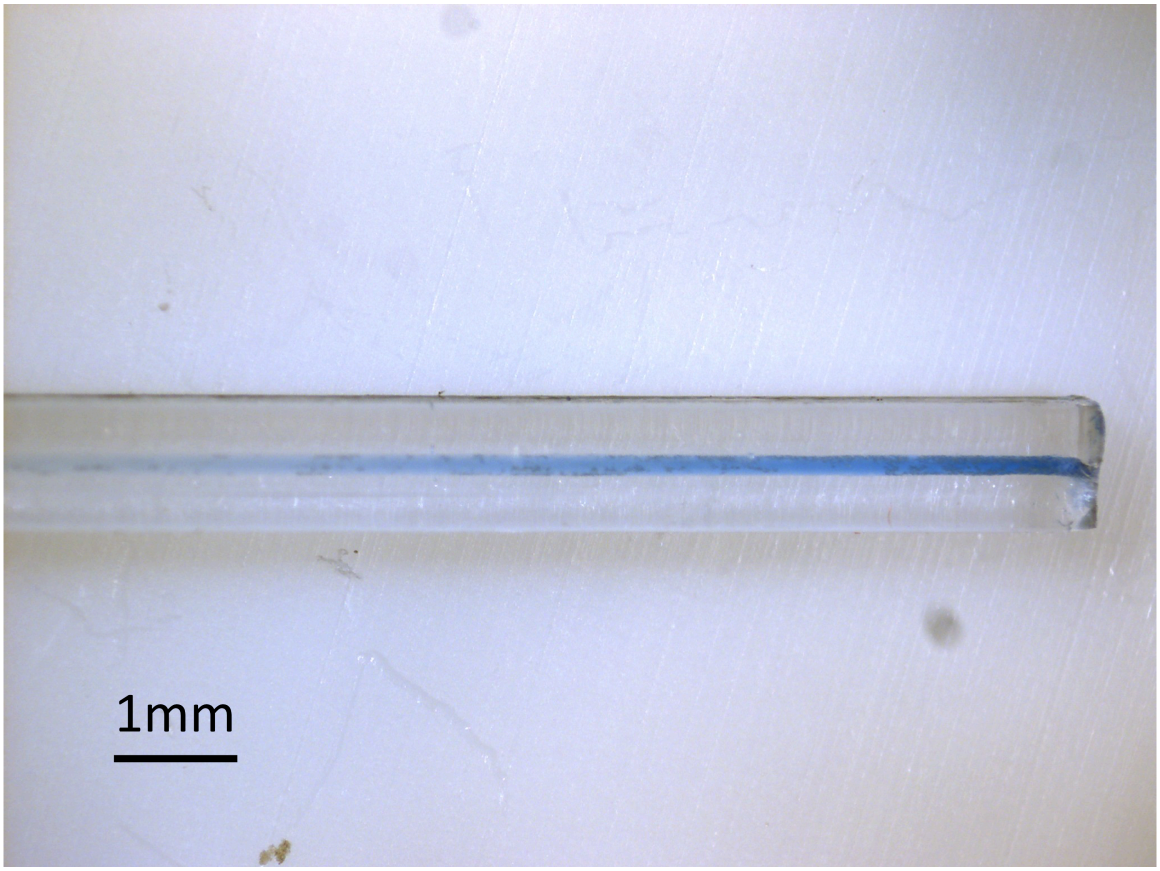

Supplementary Figure 1 :: Optical micrograph of a porous PCL core/dense COC cladding fiber with on end dipped in Sudan Blue in PC/Triethylene glycol solution for 48 hrs.

Supplementary Note 2: Viscosity data

We performed zero-shear viscosity measurement over a range of temperature using a AR2000 rheometer (TA Instruments) with a Peltier plate heater, using a 40mm diameter stainless steel 1º cone geometry. For both solutions, the minimal temperatures were chosen to be the demixing temperatures at which point viscosity data starts changing dramatically. For the pure COC, the minimal temperature corresponded to the highest range of measurable torque.

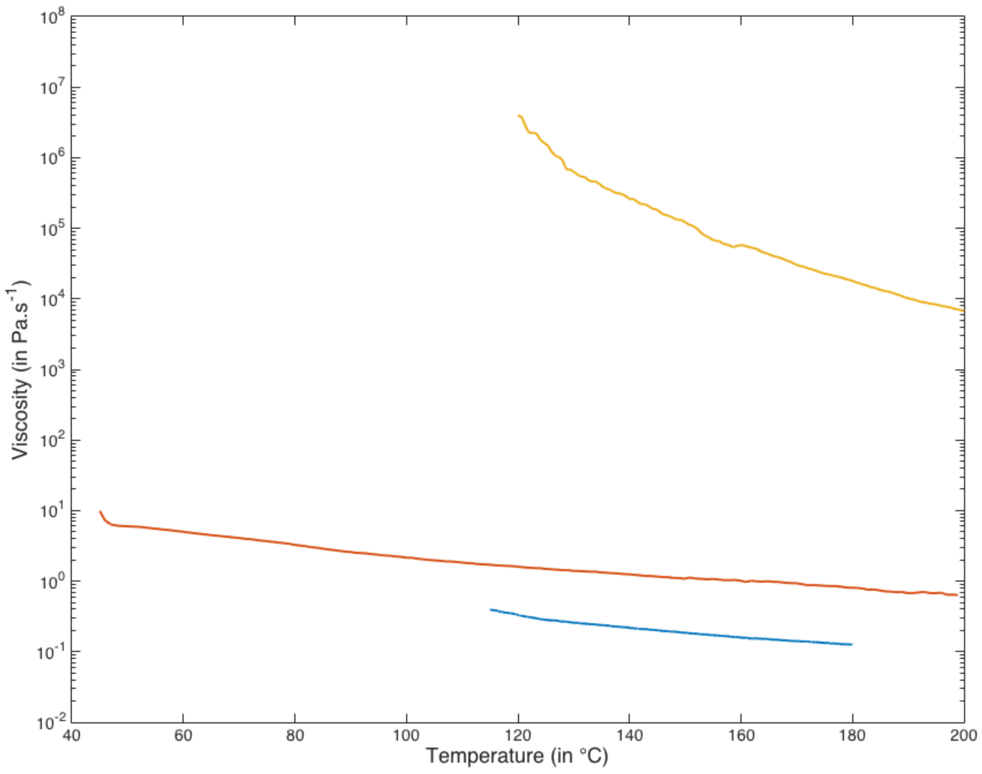

Supplementary Figure 2 :: Zero-shear viscosity versus temperature for cladding material (yellow, top curve) (COC, TOPAS 8007, yellow), 20% wt. PVdF solution in propylene carbonate (orange, middle curve), and 1:6wt. PCL solution in 1:1 wt. propylene carbonate/triethylene glycol (blue, bottom curve).

Supplementary Note 3: Ionic conductivity: axial measurement

In addition to transverse ionic conductivity measurement we demonstrated axial conductivity measurement. For this we use fibers with no CPE or metal electrodes; just a PVdF/PYR13TFSI/PC gel core. Because of the difference in aspect ratios, we could not use the sample gel composition as for the transverse measurement – this would have led to impedance values outside the range of the instrument. Therefore we increased the concentration in ionically conductive species to 1m ($mol.kg^{-1}$ of solvent) PYR13TFSI in PC, and 20% wt. PVdF in PC.

We cut the samples to desired length and immediately applied low-temperature solder (Bi-In) on the tips. We then connected the samples to the impedance analyzer with copper clips, and performed impedance measurements in the frequency range 1Hz-1MHz. We cut the samples by 20mm decrements, ramping the size from 20cm to 2cm. We took the diameter for the high frequency semicircle as the value of the bulk resistance of the electrolyte. The bulk resistance of the electrolyte scaled linearly with length and the associated conductivity value from geometric considerations was found to be 6.4±0.6 $mS.cm^{-1}$.

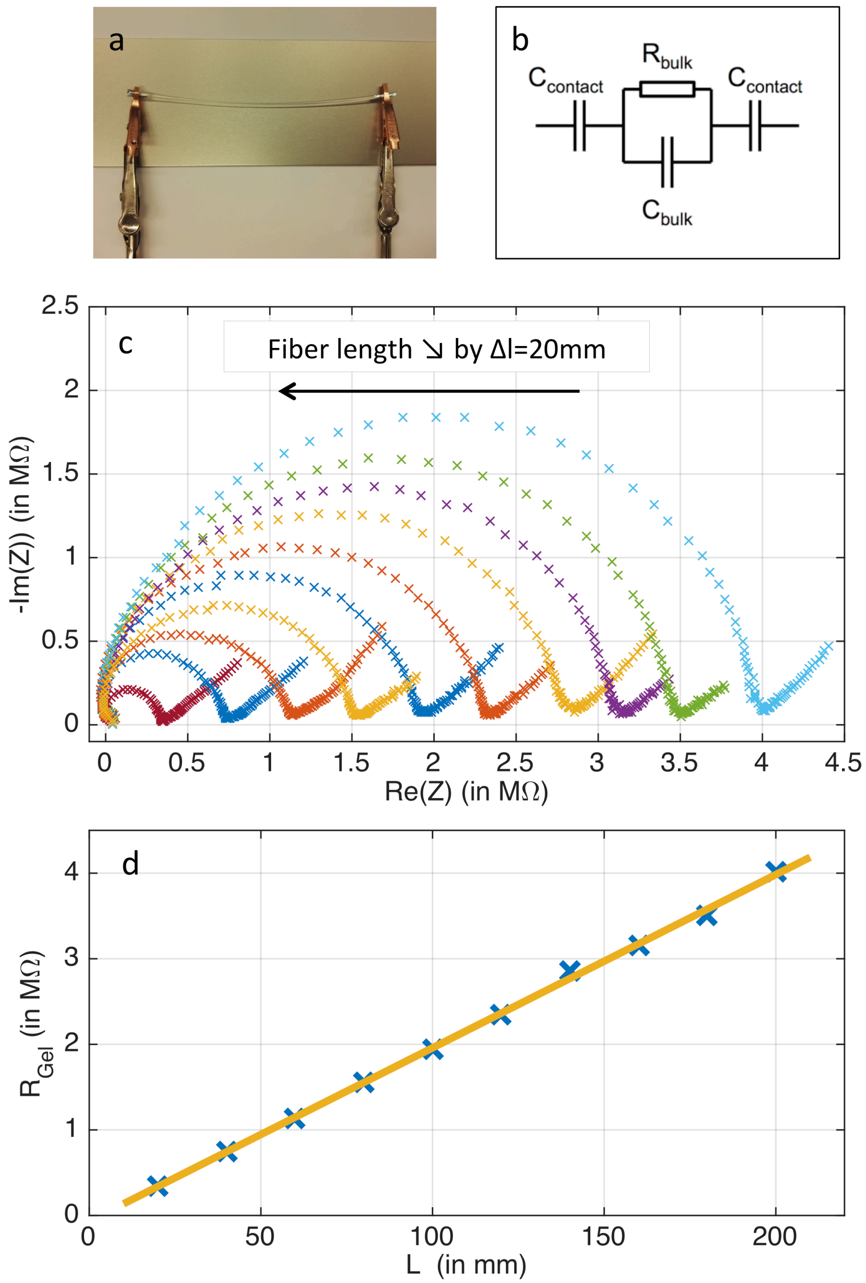

Supplementary Figure 3 :: Axial transport through impedance spectroscopy of ionic liquid-filled porous core fibers. (a) Photograph of connected COC cladding/porous PVdF core sample, with core consisting of. (b-c) Simple equivalent circuit expected from fiber samples and associated Nyquist plots. The bulk resistance of the electrolyte is read as the diameter of the high-frequency semicircle. (d) Impedance spectra for a fiber between 20cm and 2cm, sequentially cut by 20mm decrements. The bulk resistance of the electrolyte scales linearly with the fiber length.

Supplementary Note 4: Ionic conductivity: Undrawn gel electrolyte

To compare the conductivity of drawn and undrawn gel electrolyte we built to measure the conductivity of the gel electrolyte as introduced in the preform. We introduced 10^{-3} m (mol.kg^{-1} of solvent) PYR13TFSI in PC / 20% wt. PVdF in PC gels in the channels of a machined PTFE block. The extremities of the channels were tapped so that the gels could be contacted with polished stainless steel screws by direct screwing. These were then connected to the impedance analyzer to obtain Nyquist plots for the test electrolyte. The bulk resistance of the gel electrolyte was taken as the diameter of the high frequency semicircle. We obtained an ionic conductivity for the undrawn gel of 2.09 µS.cm^{-1}, from geometric considerations.

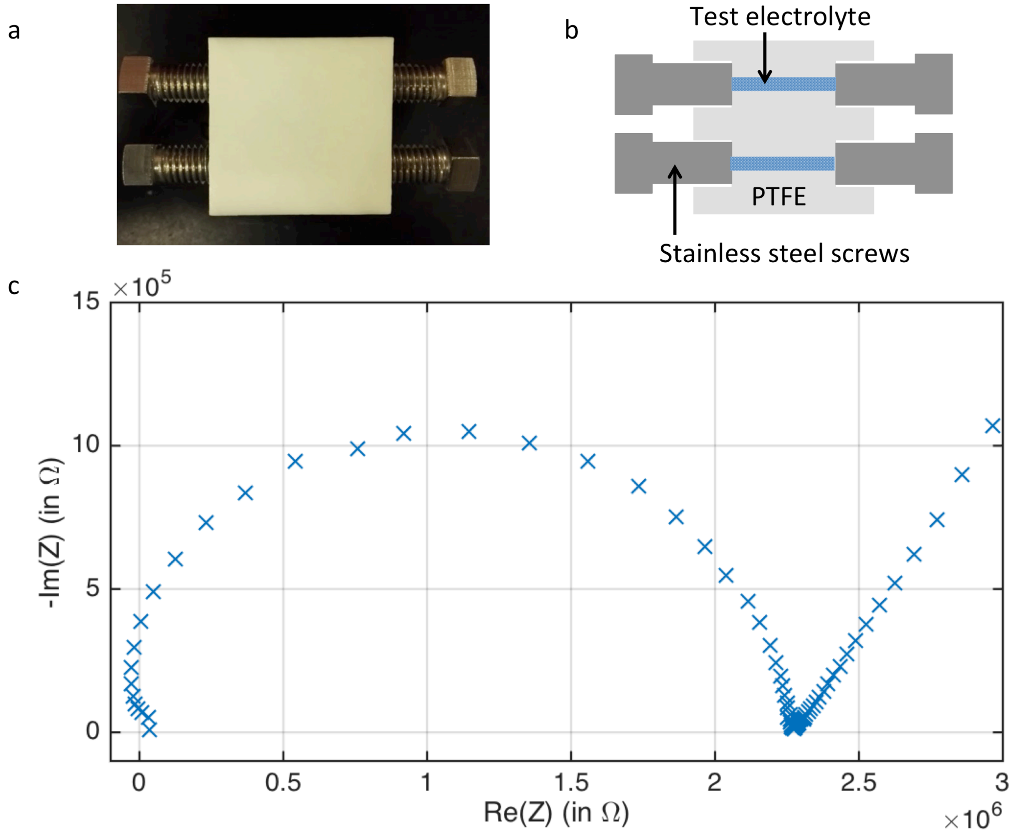

Supplementary Figure 4 :: Electrochemical impedance spectroscopy for undrawn 10^{-3} m (mol.kg^{-1} of solvent) PYR13TFSI in PC, 20% wt. PVdF in PC. (a) Photograph of measurement device. (b) Schematic cross section of measurement device. Electrolyte sample is placed in a cylindrical channel of known dimension machined in a PTFE block, and contacted with two stainless steel screws. (c) Nyquist plot obtained for undrawn gel electrolyte. Bulk resistance of the electrolyte is taken as the diameter of the high frequency semicircle.

Supplementary Discussion: Pore size versus quenching temperature

Regarding the dependence of pore size on quenching temperature for liquid-liquid polycaprolactone solution presented in Figure 3 of the manuscript, the main body of the article outlines the general framework for understanding the underlying physics of pore formation and pore size, both thermodynamically (illustration of phase diagram) and from the standpoint of kinetics (standard models for coarsening rates). This framework can be applied to other solution systems.

We here go into more details on the mechanism, and explain the necessary steps that would be required to achieve a quantitative fit of the pore size data relying only on materials parameters and processing conditions in each of the two regimes identified.

- ***Regime 1: Quenching temperature above PCL solidification***

In this regime the solution in the fiber core is in a liquid-liquid demixed state at all times of the quenching procedure. In this state, coarsening of the structure proceeds after nucleation and initial growth of the solvent-rich droplets. For polymer solutions, coarsening can commonly occur through three different mechanisms – diffusion of molecules from small to large droplets (Ostwald ripening), coalescence of droplets, or hydrodynamic flow. All these mechanisms have scaling laws of the form:

$$\langle R \rangle^{\alpha} = \langle R_0 \rangle^{\alpha} + K_{\alpha}(T)\Delta t$$

Specifically:

a. Ostwald ripening[1]: $\alpha = 3$, and $K_{\alpha} \sim \widetilde{D}\sigma\Omega^2 c_{eq}(\infty)/k_B T$

   Where $\widetilde{D}$ is the diffusivity of solvent in the polymer-rich phase, σ is the surface tension between polymer-rich phase and solvent-rich phase, Ω is the molar volume of a solvent molecule, $c_{eq}(\infty)$ is the solubility of solvent in the polymer phase for a planar polymer-rich/solvent-rich interface.

b. Droplet coalescence[2]: $\alpha = 3$, and $K_{\alpha} \sim k_B T/\eta$

   Where is the viscosity of the polymer-rich phase

c. Hydrodynamic flow[2]: $\alpha = 1$, and $K_{\alpha} \sim \sigma/\eta$

   Where is the viscosity of the polymer-rich phase and is the surface tension between the polymer-rich phase and the solvent-rich phase

For a quantitative fit of the pore size data, one would first need to identify the appropriate mechanism for the solution of interest. Standardly, this is done by conducting measurements of pore size versus time at various temperatures. Then,

one could in practice measure the required materials properties to deduce the functional $K_{\alpha}(T)$.

In practice however mechanisms a. b. and c. all occur concurrently to some degree, and different mechanisms may dominate at different times[2]. For instance, mechanisms a. and b. in general dominate over mechanism c. at early times, but there is a crossover from a./b. to c. as coarsening time increases[2]. In addition, whether a. or b. dominates in the early stage can be a function of temperature, which governs the magnitude of the prefactor K_{α}. Furthermore, because both a. and b. have the same time dependence, differentiating them requires additional measurements, but is doable in theory.

- ***Regime 2: Quenching temperature below PCL solidification***

In this regime, the solution in the fiber core is cooled from T_0 =150ºC to the bath temperature T_{bath} through convective cooling of the fiber. Coarsening of the structure only proceeds while the solution temperature is above the PCL solidification temperature – at which point the structure sets. Therefore the effective coarsening time is reduced, from Δt to $\Delta t_{eff}(T_{bath})$. The expected final pore size will be a result of coarsening over a range of temperatures:

$$\langle R \rangle^{\alpha} = \langle R_0 \rangle^{\alpha} + \int_{0}^{\Delta t_{eff}(T_{bath})} K_{\alpha}[T(t)]\, \mathrm{d}t$$

Achieving a quantitative fit depending only on materials properties and processing parameters is therefore challenging in this regime as well.

Supplementary Methods: Drawing process: parameters and data

The drawing process is performed in a custom made draw tower, with a three-zone furnace. The drawing tower enables control of the temperatures in the top, middle, and bottom zones, as well as control of the preform downfeed speed and capstan speed (or fiber pull-out speed). During the process, we monitor the fiber dimensions with a laser, as well as the instantaneous fluctuations on the fiber dimensions by computing the standard deviation over the last 10 measurements every second. In addition we also monitor the tension in the fiber. With the dimensions and tension, we compute and monitor in real time the effective stress in the fiber.

In practice during a draw, the top and bottom zone temperatures are set (for most fibers presented here $T_{\mathrm{top}} = 150$ºC and $T_{\mathrm{bot}} = 90$ºC), as well as the preform downfeed speed ($v_f = 1$mm.min^{-1}).
Therefore during the actual drawing parameters that we act one are:

- Preform draw speed, or capstan speed: v_{c}
- Middle-zone temperature: T_{mid}

while monitoring: fiber dimensions, size fluctuations, tension, and stress.

Supplementary Figures 5 and 6 present drawing data for two types of fibers: cross-shaped PVdF core, and cylindrical PVdF core used for fiber breakup demonstration. These two examples demonstrate two different types of processing which we hope will educate the reader more on this processing method.

Cross-shaped PVdF core draw [Supplementary Figure 5]
For the cross-shaped PVdF core, the objective is to maintain the inner geometry of the core and in particular the out-of-equilibrium sharp 90º angles. This example is generalizable to other structured fiber cross-sections. In the molten state, surface tension will act to round those angles with a timescale scaling linearly with the viscosity of the cladding. Therefore in order to maintain the core structure, we aim to maximize the cladding viscosity during processing, so that the timescale of the drawing process is smaller than the typical timescale for capillary rounding. This is done by processing at low temperatures. We monitor the tension and effective stress on the cladding during the drawing process so as to avoid the mechanical failure of the fiber, eg. fracture at stresses above the yield stress for COC. The sudden size peak around 45m corresponds to the entry point of the solution. The capstan speed here was controlled so as to maintain the appropriate size, aiming for a ≈300µm porous core fiber.

Cylindrical core PVdF draw for breakup [Supplementary Figure 6]
For the cylindrical core PVdF draw the objective was to obtain a cylindrical core filled with solution that would be post-processed and reheated to induce breakup and particle generation. The reheating of the fiber requires to draw at low tension and stress, otherwise the fiber can relax built-in tensile stresses when reheated and spring back into a blob as the polymer chains relax and recoil. Therefore contrary to the previous case, we aimed here for a high temperature/low tension draw. The middle zone temperature is close to 100ºC above the temperature for the processing of the cross-shaped fiber. The

result is much lower tension and stress in the fiber. The downside is that typically low fiber tension is associated with larger fluctuation dimensions; this is reinforced by the fact that fibers drawn under low tension tend to vibrate more in front of the laser setup thus causing larger fluctuations in the data. The capstan speed here was controlled again to aim for the desired fiber size. The sudden size peak around 30m corresponds to the entry point of the solution. Here the objective in terms of size was to probe the system and reach small and smaller cores, thus we continuously increased the speed to reach 10m/min of fiber drawn.

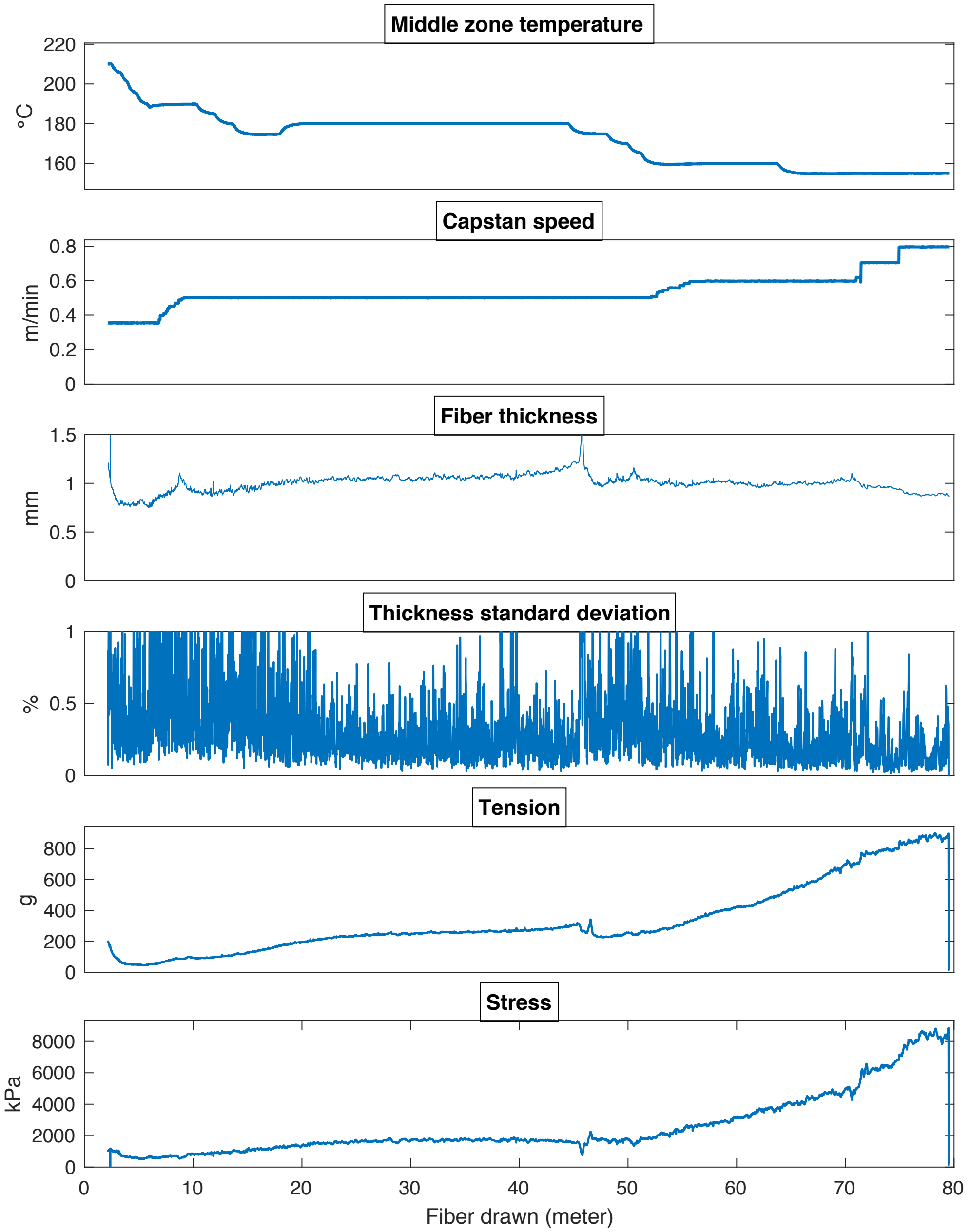

Supplementary Figure 5 :: Draw data for cross-shaped PVdF core fiber. Middle zone temperature and capstan speeds are control parameters, while fiber thickness, standard deviation, tension, and stress are measured variables.

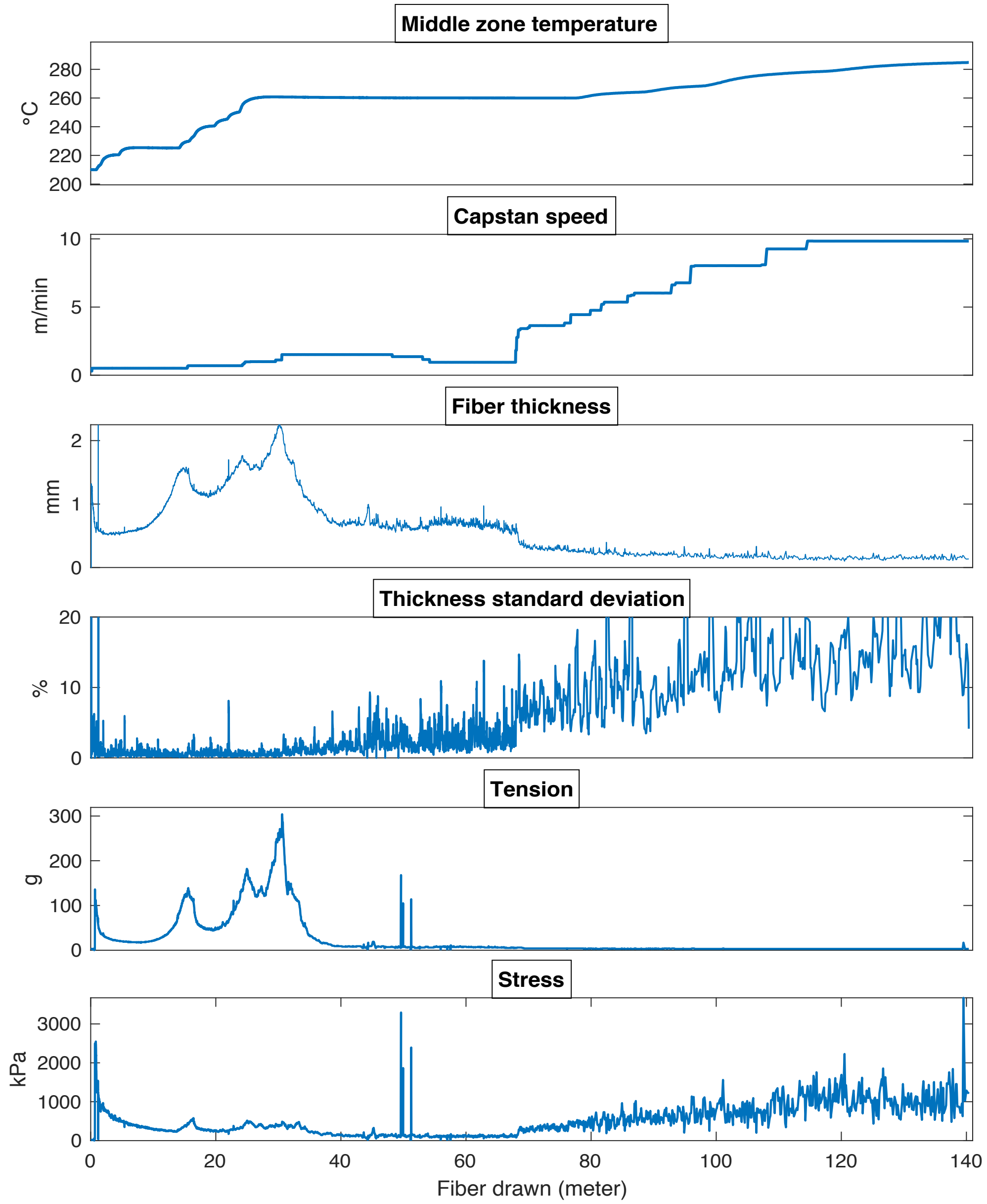

Supplementary Figure 6 :: Draw data for cylindrical PVdF core fiber for breakup. Middle zone temperature and capstan speeds are control parameters, while fiber thickness, standard deviation, tension, and stress are measured variables.

Supplementary References

1. Balluffi, R. W., Allen, S. M. & Carter, W. C. *Kinetics of Materials*. (John Wiley & Sons, Inc., 2005). doi:10.1002/0471749311
2. Song, S. & Torkelson, J. M. Coarsening Effects on Microstructure Formation in Isopycnic Polymer Solutions and Membranes Produced via Thermally Induced Phase Separation. *Macromolecules* **27,** 6389–6397 (1994).
